# Supplementary material for: The impact of Mendelian sleep and circadian genetic variants in a population setting
Source: PLoS Genet. 2022 Sep 22;18(9):e1010356. doi: 10.1371/journal.pgen.1010356 (PMC9499244; doi:10.1371/journal.pgen.1010356)
Supplement: S1 Table — (DOCX) [file pgen.1010356.s001.docx]

**S1 Table.** Summary of twelve variants previously reported to be causal for Mendelian sleep and circadian conditions, including the variant frequencies catalogued in gnomAD.

| **PubMed ID** | **Gene** | **Variant** | **CHR:BP^a^** | **rsID^b^** | **Trait** | **gnomAD AAF^f^** | **gnomAD EAF^g^** |
| --- | --- | --- | --- | --- | --- | --- | --- |
| 33065013 | *GRM1* | S458A | 6:146352435 | rs151255685 | FNSS^c^ | 2x10^-4^ | 2x10^-4^ |
| 33065013 | *GRM1* | R889W | 6:146426563 | rs768023437 | FNSS^c^ | 3x10^-5^ | 6x10^-5^ |
| 31619542 | *NPSR1* | Y206H | 7:34827538 | rs1406844918 | FNSS^c^ | 4x10^-6^ | 9x10^-6^ |
| 31473062 | *ADRB1* | A187V | 10:114044692 | rs776439595 | FNSS^c^ | 4x10^-5^ | 8x10^-5^ |
| 19679812 | *DEC2/BHLHE41* | P384R | 12:26122364 | rs121912617 | FNSS^c^ | 3x10^-5^ | 5x10^-5^ |
| 28388406 | *CRY1* | c.1657+3A>C | 12:106992962 | rs184039278 | DSPD^d^ | 4x10^-3^ | 5x10^-3^ |
| 26903630 | *PER3* | P415A | 1:7809893 | rs150812083 | FASP^e^ | 6x10^-3^ | 6x10^-3^ |
| 26903630 | *PER3* | H417R | 1:7809900 | rs139315125 | FASP^e^ | 6x10^-3^ | 6x10^-3^ |
| 11232563 | *PER2* | PER2S662G | 2:238257003 | rs121908635 | FASP^e^ | - | - |
| 27529127 | *CRY2* | A260T | 11:45867648 | rs201220841 | FASP^e^ | 6x10^-5^ | 1x10^-4^ |
| 31138685 | *TIMELESS* | R1081X | 12:56418347 | rs1465092391 | FASP^e^ | 8x10^-6^ | 2x10^-5^ |
| 15800623 | *CSNK1D* | T44A | 17:82265743 | rs104894561 | FASP^e^ | 4x10^-6^ | 9x10-^6^ |

^a^chromosome and base-pair position defined by the Genome Reference Consortium Human Build 38; ^b^reference SNP (rs) cluster identifier of variant; ^c^familial natural short sleep; ^d^delayed sleep phase disorder; ^e^familial advanced sleep phase; ^f^allele frequency of minor allele across all ancestries catalogued in gnomAD (version 2.1). ^g^allele frequency of minor allele within samples of European ancestry (excluding Finnish) catalogued in gnomAD (version 2.1).
