# Supplementary material for: The impact of Mendelian sleep and circadian genetic variants in a population setting
Source: PLoS Genet. 2022 Sep 22;18(9):e1010356. doi: 10.1371/journal.pgen.1010356 (PMC9499244; doi:10.1371/journal.pgen.1010356)
Supplement: S2 Table — Genotype counts are based on availability of sleep characteristics relevant to each gene. (DOCX) [file pgen.1010356.s002.docx]

**S2 Table.** Maximum genotype counts for 12 previously reported monogenic causes of sleep and circadian conditions in unrelated individuals of European ancestry from the UK Biobank, FINRISK & Health 2000-2011 and MESA studies. Genotype counts are based on availability of sleep characteristics relevant to each gene.

|  |  |  | **UK Biobank** | | | **FINRISK & Health 2000-2011** | | | **MESA** | | |
| --- | --- | --- | --- | --- | --- | --- | --- | --- | --- | --- | --- |
| **Gene / Variant** | **Trait** | **REF/ALT^d^** | **REF/REF^e^** | **REF/ALT^f^** | **ALT/ALT^g^** | **REF/REF^e^** | **REF/ALT^f^** | **ALT/ALT^g^** | **REF/REF^e^** | **REF/ALT^f^** | **ALT/ALT^g^** |
| *GRM1* / S458A | FNSS^a^ | T/G | 169,451 | 67 | - | 5,927 | <5 | 0 | - | - | - |
| *GRM1* / R889W | FNSS^a^ | A/T | 169,513 | 3 | - | - | - | - | - | - | - |
| *NPSR1* / Y206H | FNSS^a^ | T/C | - | - | - | - | - | - | - | - | - |
| *ADRB1* / A187V | FNSS^a^ | C/T | 169,450 | 69 | - | - | - | - | - | - | - |
| *DEC2/BHLHE41* / P384R | FNSS^a^ | G/C | 169,500 | 10 | - | - | - | - | 1,993 | 22 | - |
| *CRY1* / c.1657+3A>C | DSPD^b^ | T/G | 168,586 | 1,480 | 9 | 2,838 | <5 | 0 | - | - | - |
| *PER3* / P415A | FASP^c^ | C/G | 168,500 | 1,565 | 7 | 2,734 | 149 | <5 | 2,003 | 12 | - |
| *PER3* / H417R | FASP^c^ | A/G | 168,500 | 1,567 | 7 | 2,734 | 149 | <5 | 2,003 | 12 | - |
| *PER2* / PER2S662G | FASP^c^ | A/G | - | - | - | - | - | - | - | - | - |
| *CRY2* / A260T | FASP^c^ | G/A | 170,038 | 38 | - | - | - | - | - | - | - |
| *TIMELESS* / R1081X | FASP^c^ | G/A | 170,064 | 5 | - | - | - | - | - | - | - |
| *CSNK1D* / T44A | FASP^c^ | T/C | 170,075 | 1 | - | - | - | - | - | - | - |

^a^familial natural short sleep; ^b^delayed sleep phase disorder; ^c^familial advanced sleep phase; ^d^reference allele / alternate allele; ^e^number of homozygous carriers for reference allele; ^f^number of heterozygous carriers; ^g^number of homozygous carriers for alternate allele.
