# Supplementary material for: The impact of Mendelian sleep and circadian genetic variants in a population setting
Source: PLoS Genet. 2022 Sep 22;18(9):e1010356. doi: 10.1371/journal.pgen.1010356 (PMC9499244; doi:10.1371/journal.pgen.1010356)
Supplement: S3 Table — (DOCX) [file pgen.1010356.s003.docx]

**S3 Table.** Summary statistics of self-reported sleep duration in the UK Biobank (Field 1160) for carriers of variants previously described as causal for familial natural short sleep.

| **Gene** | **Variant** | **REF/ALT^a^** | **Study** | **Genotype** | **N** | **Minimum** | **Maximum** | **Mean** | **SD^b^** | **P^c^** |
| --- | --- | --- | --- | --- | --- | --- | --- | --- | --- | --- |
| *ADRB1* | A187V | C/T | UKB | C/C | 166,291 | 1 | 12 | 7.17 | 1.07 | 0.615 |
|  |  |  |  | C/T | 69 | 5 | 12 | 7.10 | 1.03 |  |
| *DEC2/*  *BHLHE41* | P384R | G/C | UKB | G/G | 166,283 | 1 | 12 | 7.17 | 1.07 | 0.694 |
|  |  |  |  | G/C | 10 | 5 | 9 | 7.30 | 1.06 |  |
| *GRM1* | S458A | T/G | UKB | T/T | 166,290 | 1 | 12 | 7.17 | 1.07 | 0.807 |
|  |  |  |  | T/G | 67 | 4 | 10 | 7.13 | 1.15 |  |
|  |  |  | FINRISK/  Health 2000-2011 | T/T | 12027 | 3 | 15 | 7.4 | 1.14 | 0.390 |
|  |  |  |  | T/G | 6 | 5 | 9 | 7 | 1.41 |  |
|  | A889T | A/T | UKB | A/A | 166,288 | 1 | 12 | 7.17 | 1.07 | 0.179 |
|  |  |  |  | A/T | 3 | 8 | 8 | 8.00 | 0.00 |  |

^a^Reference and alternate allele relative to reference genome; ^b^Standard Deviation; ^c^P-value derived from 2-sided t-test.
