# Supplementary material for: The impact of Mendelian sleep and circadian genetic variants in a population setting
Source: PLoS Genet. 2022 Sep 22;18(9):e1010356. doi: 10.1371/journal.pgen.1010356 (PMC9499244; doi:10.1371/journal.pgen.1010356)
Supplement: S4 Table — Data unavailable for self-reported sleep of ≤5 hours, ≤4 hours and 4–6 hours in the Finnish study. (DOCX) [file pgen.1010356.s004.docx]

**S4 Table.** Summary statistics of dichotomised self-reported sleep data in the UK Biobank for carriers of variants previously described as causal for familial natural short sleep. Data unavailable for self-reported sleep of ≤5 hours, ≤4 hours and 4-6 hours in the Finnish study.

|  |  |  |  |  | **Short Sleep ( ≤6 hours)** | | | | **Short Sleep (≤5 hours)** | | | | **Short Sleep (≤4 hours)** | | | | **Short Sleep (4 to 6 hours)** | | | |
| --- | --- | --- | --- | --- | --- | --- | --- | --- | --- | --- | --- | --- | --- | --- | --- | --- | --- | --- | --- | --- |
| **Gene** | **Variant** | **REF/**  **ALT^a^** | **Study** | **Genotype** | **%**  **cases** | **N**  **cases** | **N**  **controls** | **P^b^** | **%**  **cases** | **N**  **cases** | **N**  **controls** | **P^b^** | **%**  **cases** | **N**  **cases** | **N**  **controls** | **P^b^** | **%**  **cases** | **N**  **cases** | **N**  **controls** | **P^b^** |
| *ADRB1* | A187V | C/T | UKB | C/C | 23.74 | 39,484 | 126,807 | 1.000 | 5.08 | 8,446 | 157,845 | 1.000 | 1.01 | 1,679 | 164,612 | 1.000 | 23.56 | 39,176 | 127,115 | 1.000 |
|  |  |  |  | C/T | 23.19 | 16 | 53 |  | 4.35 | 3 | 66 |  | 0.00 | 0 | 69 |  | 23.19 | 16 | 53 |  |
| *DEC2/*  *BHLHE41* | P384R | G/C | UKB | G/G | 23.74 | 39,483 | 126,800 | 0.469 | 5.08 | 8,446 | 157,837 | 0.406 | 1.01 | 1,679 | 164,604 | 1.000 | 23.56 | 39,175 | 127,108 | 0.470 |
|  |  |  |  | G/C | 10.00 | 1 | 9 |  | 10.00 | 1 | 9 |  | 0.00 | 0 | 10 |  | 10.00 | 1 | 9 |  |
| *GRM1* | S458A | T/G | UKB | T/T | 23.74 | 39,484 | 126,806 | 0.389 | 5.08 | 8,446 | 157,844 | 0.393 | 1.01 | 1,679 | 164,611 | 0.494 | 23.56 | 39,176 | 127,114 | 0.387 |
|  |  |  |  | T/G | 28.36 | 19 | 48 |  | 7.46 | 5 | 62 |  | 1.49 | 1 | 66 |  | 28.36 | 19 | 48 |  |
|  |  |  | FINRISK/  Health 2000-2011 | T/T | 17.88 | 2,151 | 9,876 | 0.290 | NA | NA | NA | NA | NA | NA | NA | NA | NA | NA | NA | NA |
|  |  |  |  | T/G | 33.33 | <5 | <5 |  | NA | NA | NA |  | NA | NA | NA |  | NA | NA | NA |  |
|  | A889T | A/T | UKB | A/A | 23.75 | 39,485 | 126,803 | 1.000 | 5.08 | 8,446 | 157,842 | 1.000 | 1.01 | 1,679 | 164,609 | 1.000 | 23.56 | 39,177 | 127,111 | 1.000 |
|  |  |  |  | A/T | 0.00 | 0 | 3 |  | 0.00 | 0 | 3 |  | 0.00 | 0 | 3 |  | 0.00 | 0 | 3 |  |

^a^Reference and alternate allele relative to reference genome; ^b^P-value derived from 2-sided Fisher’s exact-test.
