# Supplementary material for: The impact of Mendelian sleep and circadian genetic variants in a population setting
Source: PLoS Genet. 2022 Sep 22;18(9):e1010356. doi: 10.1371/journal.pgen.1010356 (PMC9499244; doi:10.1371/journal.pgen.1010356)
Supplement: S5 Table — No carriers of the GRM1 A889T variant remained among individuals from UK Biobank who had worn an accelerometer. (DOCX) [file pgen.1010356.s005.docx]

**S5 Table.** Summary statistics of accelerometer-derived estimates of sleep duration in UK Biobank for carriers of variants previously described as casual for familial natural short sleep. No carriers of the *GRM1* A889T variant remained among individuals from UK Biobank who had worn an accelerometer.

|  |  |  |  | **All Nights (hours)** | | | | | **Weeknights (hours)** | | | | | **Weekend Nights (hours)** | | | | |
| --- | --- | --- | --- | --- | --- | --- | --- | --- | --- | --- | --- | --- | --- | --- | --- | --- | --- | --- |
| **Gene** | **Variant** | **REF/**  **ALT^a^** | **Genotype** | **N** | **Min^b^** | **Max^c^** | **Mean (SD^d^)** | **P^e^** | **N** | **Min^b^** | **Max^c^** | **Mean (SD^d^)** | **P^e^** | **N** | **Min^b^** | **Max^c^** | **Mean (SD^d^)** | **P^e^** |
| *ADRB1* | A187V | C/T | C/C | 34,168 | 1.63 | 11.87 | 7.30 (0.86) | 0.197 | 34,134 | 1.35 | 11.87 | 7.25 (0.92) | 0.178 | 33,271 | 1.01 | 11.63 | 7.44 (1.09) | 0.500 |
|  |  |  | C/T | 15 | 6.89 | 8.83 | 7.59 (0.49) |  | 15 | 6.90 | 8.73 | 7.57 (0.47) |  | 15 | 6.50 | 9.03 | 7.63 (0.81) |  |
| *DEC2/*  *BHLHE41* | P384R | G/C | G/G | 34,167 | 1.63 | 11.87 | 7.30 (0.86) | 0.624 | 34,133 | 1.35 | 11.87 | 7.25 (0.92) | 0.484 | 33,270 | 1.01 | 11.63 | 7.44 (1.09) | 0.858 |
|  |  |  | G/C | 4 | 6.26 | 8.25 | 7.51 (0.92) |  | 4 | 6.25 | 8.29 | 7.57 (0.92) |  | 4 | 6.27 | 8.60 | 7.35 (1.06) |  |
| *GRM1* | S458A | T/G | T/T | 34,166 | 1.63 | 11.87 | 7.30 (0.86) | 0.915 | 34,132 | 1.35 | 11.87 | 7.25 (0.92) | 0.590 | 33,269 | 1.01 | 11.63 | 7.44 (1.09) | 0.331 |
|  |  |  | T/G | 10 | 6.48 | 8.30 | 7.33 (0.76) |  | 10 | 6.55 | 8.34 | 7.40 (0.79) |  | 10 | 5.07 | 8.87 | 7.11 (1.14) |  |
| *GRM1* | A889T | A/T | A/A | 34,167 | 1.63 | 11.87 | 7.30 (0.86) | NA | 34,133 | 1.35 | 11.87 | 7.25 (0.92) | NA | 33,270 | 1.01 | 11.63 | 7.44 (1.09) | NA |
|  |  |  | A/T | 0 | NA | NA | NA |  | 0 | NA | NA | NA |  | 0 | NA | NA | NA |  |

^a^Reference and alternate allele relative to reference genome; ^b^Minimum; ^c^Maximum; ^d^Standard deviation; ^e^P-value from 2-sided t-test.
