# Supplementary material for: The impact of Mendelian sleep and circadian genetic variants in a population setting
Source: PLoS Genet. 2022 Sep 22;18(9):e1010356. doi: 10.1371/journal.pgen.1010356 (PMC9499244; doi:10.1371/journal.pgen.1010356)
Supplement: S6 Table — Data on being “more or definitely a morning person” unavailable in the Finnish studies. (DOCX) [file pgen.1010356.s006.docx]

**S6 Table.** Summary statistics of “Morningness” across genotype groups for variants previously reported as causal for familial advanced sleep phase. Data on being “more or definitely a morning person” unavailable in the Finnish studies.

|  |  |  |  | **Definitely a morning person** | | | | **More or definitely a morning person** | | | |
| --- | --- | --- | --- | --- | --- | --- | --- | --- | --- | --- | --- |
| **Gene** | **Variant** | **Study** | **Genotype** | **%**  **cases** | **N**  **cases** | **N**  **controls** | **P^a^** | **%**  **cases** | **N**  **cases** | **N**  **controls** | **P^a^** |
| *PER3* | P415A | UKB | C/C | 23.77 | 39,655 | 127,177 | <0.0001 | 56.24 | 93,827 | 73,005 | <0.0001 |
|  |  |  | C/G | 29.58 | 463 | 1,102 |  | 62.94 | 985 | 580 |  |
|  |  |  | G/G | 42.86 | 3 | 4 |  | 71.43 | 5 | 2 |  |
|  |  | FINRISK/  Health 2000-2011 | C/C | 22.4 | 613 | 2,121 | 1.000 | NA | NA | NA | NA |
|  |  |  | C/G | 22.8 | 34 | 115 |  | NA | NA | NA |  |
|  |  |  | G/G | 0.0 | 0 | <5 |  | NA | NA | NA |  |
|  | H417R | UKB | A/A | 23.77 | 39,655 | 127,177 | <0.0001 | 56.24 | 93,827 | 73,005 | <0.0001 |
|  |  |  | A/G | 29.55 | 463 | 1,104 |  | 62.86 | 985 | 582 |  |
|  |  |  | G/G | 42.86 | 3 | 4 |  | 71.43 | 5 | 2 |  |
|  |  | FINRISK/  Health 2000-2011 | A/A | 22.4 | 613 | 2,121 | 1.000 | NA | NA | NA | NA |
|  |  |  | A/G | 22.8 | 34 | 115 |  | NA | NA | NA |  |
|  |  |  | G/G | 0.0 | 0 | <5 |  | NA | NA | NA |  |
| *CRY2* | A260T | UKB | G/G | 23.77 | 39,655 | 127,179 | 0.340 | 56.24 | 93,827 | 73,007 | 0.327 |
|  |  |  | G/A | 15.79 | 6 | 32 |  | 47.37 | 18 | 20 |  |
| *TIMELESS* | R1081X | UKB | G/G | 23.77 | 39,652 | 127,175 | 1.000 | 56.24 | 93,822 | 73,005 | 0.175 |
|  |  |  | G/A | 20.00 | 1 | 4 |  | 20 | 1 | 4 |  |

^a^P-value derived from 2-sided Fisher’s exact test. Homozygous carriers for a *PER3* variant allele were combined with heterozygous carriers prior to performing Fisher’s exact test.
