# Supplementary material for: The impact of Mendelian sleep and circadian genetic variants in a population setting
Source: PLoS Genet. 2022 Sep 22;18(9):e1010356. doi: 10.1371/journal.pgen.1010356 (PMC9499244; doi:10.1371/journal.pgen.1010356)
Supplement: S7 Table — (DOCX) [file pgen.1010356.s007.docx]

**S7 Table.** Summary statistics of L5-midpoint timing estimated from accelerometer data in UK Biobank across genotype groups for variants previously reported as causal for familial advanced sleep phase.

|  |  |  | **Average for All Nights** | | | | | **Average for Weeknights** | | | | | **Average for Weekend Nights** | | | | |
| --- | --- | --- | --- | --- | --- | --- | --- | --- | --- | --- | --- | --- | --- | --- | --- | --- | --- |
| **Gene** | **Variant** | **Genotype** | **N** | **Min^a^** | **Max^b^** | **Mean (SD^c^)** | **P^d^** | **N** | **Min^a^** | **Max^b^** | **Mean (SD^c^)** | **P^d^** | **N** | **Min^a^** | **Max^b^** | **Mean (SD^c^)** | **P^d^** |
| *PER3* | P415A | C/C | 33,998 | 23.08 | 31.51 | 27.32 (0.99) | 0.053 | 33,964 | 22.79 | 31.80 | 27.29 (1.05) | 0.053 | 32,636 | 21.50 | 33.32 | 27.42 (1.36) | 0.076 |
|  |  | C/G | 338 | 21.34 | 31.43 | 27.21 (1.05) |  | 337 | 20.09 | 31.53 | 27.18 (1.16) |  | 325 | 13.00 | 30.92 | 27.28 (1.57) |  |
|  |  | G/G | 1 | 28.01 | 28.01 | 28.01 |  | 1 | 27.30 | 27.30 | 27.30 |  | 1 | 29.44 | 29.44 | 29.44 |  |
|  | H417R | A/A | 33,997 | 23.08 | 31.51 | 27.32 (0.99) | 0.060 | 33,963 | 22.79 | 31.80 | 27.29 (1.05) | 0.060 | 32,635 | 21.50 | 33.32 | 27.42 (1.36) | 0.076 |
|  |  | A/G | 339 | 21.34 | 31.43 | 27.21 (1.05) |  | 338 | 20.09 | 31.53 | 27.19 (1.16) |  | 325 | 13.00 | 30.92 | 27.28 (1.57) |  |
|  |  | G/G | 1 | 28.01 | 28.01 | 28.01 |  | 1 | 27.30 | 27.30 | 27.30 |  | 1 | 29.44 | 29.44 | 29.44 |  |
| *CRY2* | A260T | G/G | 33,998 | 23.08 | 31.51 | 27.32 (0.99) | 0.092 | 33,964 | 22.79 | 31.80 | 27.29 (1.05) | 0.311 | 32,636 | 21.50 | 33.32 | 27.42 (1.36) | 0.031 |
|  |  | G/A | 4 | 27.32 | 28.67 | 28.15 (0.58) |  | 4 | 26.91 | 28.67 | 27.82 (0.73) |  | 3 | 28.98 | 29.31 | 29.12 (0.17) |  |
| *TIMELESS* | R1081X | G/G | 33,997 | 23.08 | 31.51 | 27.32 (0.99) | NA | 33,963 | 22.79 | 31.80 | 27.29 (1.05) | NA | 32,635 | 21.50 | 33.32 | 27.42 (1.36) | NA |
|  |  | G/A | 0 | NA | NA | NA |  | 0 | NA | NA | NA |  | 0 | NA | NA | NA |  |

^a^Minimum; ^b^Maximum; ^c^Standard Deviation; ^d^P-value from 2-sided t-test. Homozygous carriers for a *PER3* variant allele were combined with heterozygous carriers prior to performing t*-*tests.
