# Supplementary material for: The impact of Mendelian sleep and circadian genetic variants in a population setting
Source: PLoS Genet. 2022 Sep 22;18(9):e1010356. doi: 10.1371/journal.pgen.1010356 (PMC9499244; doi:10.1371/journal.pgen.1010356)
Supplement: S8 Table — (DOCX) [file pgen.1010356.s008.docx]

**S8 Table.** Summary statistics of sleep-midpoint estimated from accelerometer data in UK Biobank and MESA across genotype groups for variants previously reported as causal for familial advanced sleep phase.

|  |  |  |  | **Average for All Nights** | | | | | **Average for Weeknights** | | | | | **Average for Weekend Nights** | | | | |
| --- | --- | --- | --- | --- | --- | --- | --- | --- | --- | --- | --- | --- | --- | --- | --- | --- | --- | --- |
| **Gene** | **Variant** | **Study** | **Genotype** | **N** | **Min^a^** | **Max^b^** | **Mean (SD^c^)** | **P^d^** | **N** | **Min^a^** | **Max^b^** | **Mean (SD^c^)** | **P^d^** | **N** | **Min^a^** | **Max^b^** | **Mean (SD^c^)** | **P^d^** |
| *PER3* | P415A | UKB | C/C | 33,908 | 23.37 | 30.59 | 27.01 (0.85) | 0.014 | 33,874 | 23.01 | 30.59 | 26.92 (0.91) | 0.005 | 33,022 | 21.69 | 32.68 | 27.27 (1.25) | 0.256 |
|  |  |  | C/G | 338 | 19.45 | 29.64 | 26.90 (0.96) |  | 337 | 19.22 | 29.42 | 26.77 (1.03) |  | 333 | 18.92 | 30.99 | 27.19 (1.48) |  |
|  |  |  | G/G | 1 | 27.60 | 27.60 | 27.60 |  | 1 | 27.39 | 27.39 | 27.39 |  | 1 | 28.12 | 28.12 | 28.12 |  |
|  |  | MESA | C/C | 1,925 | 13.15 | 34.92 | 27.05 (2.16) | 0.900 | 1,924 | 13.00 | 34.69 | 27.02 (2.02) | 0.888 | 1,915 | 13.00 | 33.92 | 27.46 (1.7) | 0.123 |
|  |  |  | C/G | 10 | 26.12 | 27.98 | 26.96 (0.67) |  | 10 | 26.08 | 28.64 | 27.11 (0.84) |  | 10 | 25.46 | 27.80 | 26.63 (0.66) |  |
|  | H417R | UKB | A/A | 33,907 | 23.37 | 30.59 | 27.01 (0.85) | 0.018 | 33,873 | 23.01 | 30.59 | 26.92 (0.91) | 0.006 | 33,021 | 21.69 | 32.68 | 27.27 (1.25) | 0.272 |
|  |  |  | A/G | 339 | 19.45 | 29.64 | 26.90 (0.96) |  | 338 | 19.22 | 29.42 | 26.78 (1.03) |  | 334 | 18.92 | 30.99 | 27.19 (1.48) |  |
|  |  |  | G/G | 1 | 27.60 | 27.60 | 27.60 |  | 1 | 27.39 | 27.39 | 27.39 |  | 1 | 28.12 | 28.12 | 28.12 |  |
|  |  | MESA | A/A | 1,925 | 13.15 | 34.92 | 27.05 (2.16) | 0.900 | 1,924 | 13.00 | 34.69 | 27.02 (2.02) | 0.888 | 1,915 | 13.00 | 33.92 | 27.46 (1.7) | 0.123 |
|  |  |  | A/G | 10 | 26.12 | 27.98 | 26.96 (0.67) |  | 10 | 26.08 | 28.64 | 27.11 (0.84) |  | 10 | 25.46 | 27.80 | 26.63 (0.66) |  |
| *CRY2* | A260T | UKB | G/G | 33,908 | 23.37 | 30.59 | 27.01 (0.85) | 0.040 | 33,874 | 23.01 | 30.59 | 26.92 (0.91) | 0.142 | 33,022 | 21.69 | 32.68 | 27.27 (1.25) | 0.005 |
|  |  |  | G/A | 4 | 26.63 | 29.34 | 27.88 (1.14) |  | 4 | 26.63 | 28.72 | 27.59 (0.91) |  | 3 | 28.38 | 30.89 | 29.29 (1.39) |  |
| *TIMELESS* | R1081X | UKB | G/G | 33,907 | 23.37 | 30.59 | 27.01 (0.85) | NA | 33,873 | 23.01 | 30.59 | 26.92 (0.91) | NA | 33,021 | 21.69 | 32.68 | 27.27 (1.25) | NA |
|  |  |  | G/A | 0 | NA | NA | NA |  | 0 | NA | NA | NA |  | 0 | NA | NA | NA |  |

^a^Minimum; ^b^Maximum; ^c^Standard Deviation; ^d^P-value from 2-sided t-test. Homozygous carriers for a *PER3* variant allele were combined with heterozygous carriers prior to performing t*-*tests.
