# Supplementary material for: The impact of Mendelian sleep and circadian genetic variants in a population setting
Source: PLoS Genet. 2022 Sep 22;18(9):e1010356. doi: 10.1371/journal.pgen.1010356 (PMC9499244; doi:10.1371/journal.pgen.1010356)
Supplement: S9 Table — Data on being “more or definitely an evening person” unavailable in the Finnish studies. (DOCX) [file pgen.1010356.s009.docx]

**S9 Table.** Summary statistics of “eveningness” across genotype groups for variants previously reported as causal for delayed sleep phase. Data on being “more or definitely an evening person” unavailable in the Finnish studies.

|  |  |  |  | **Definitely an evening person** | | | | **More or definitely an evening person** | | | |
| --- | --- | --- | --- | --- | --- | --- | --- | --- | --- | --- | --- |
| **Gene** | **Variant** | **Study** | **Genotype** | **%**  **cases** | **N**  **cases** | **N**  **controls** | **P^a^** | **%**  **cases** | **N**  **cases** | **N**  **controls** | **P^a^** |
| *CRY1* | c.1657+3A>C | UKB | T/T | 7.92 | 13,212 | 153,621 | 0.003 | 33.48 | 55,862 | 110,971 | 0.001 |
|  |  |  | T/G | 10.07 | 149 | 1,331 |  | 37.91 | 561 | 919 |  |
|  |  |  | G/G | 11.11 | 1 | 8 |  | 22.22 | 2 | 7 |  |
|  |  | FINRISK/  Health 2000-2011 | T/T | 10 | 284 | 2,554 | 1.000 | NA | NA | NA | NA |
|  |  |  | T/G | 0 | 0 | <5 |  | NA | NA | NA |  |

^a^P-value from 2-sided Fisher’s exact test. Homozygous carriers for the *CRY1* variant in the UK Biobank were combined with heterozygous carriers prior to performing Fisher’s exact tests.
