# Supplementary material for: The impact of Mendelian sleep and circadian genetic variants in a population setting
Source: PLoS Genet. 2022 Sep 22;18(9):e1010356. doi: 10.1371/journal.pgen.1010356 (PMC9499244; doi:10.1371/journal.pgen.1010356)
Supplement: S10 Table — (DOCX) [file pgen.1010356.s010.docx]

**S10 Table.** Summary statistics of L5-midpoint timing estimated from accelerometer data in UK Biobank a across genotype groups for variants previously reported as causal for delayed sleep phase.

|  |  |  | **Average for All Nights** | | | | | **Average for Weeknights** | | | | | **Average for Weekend Nights** | | | | |
| --- | --- | --- | --- | --- | --- | --- | --- | --- | --- | --- | --- | --- | --- | --- | --- | --- | --- |
| **Gene** | **Variant** | **Genotype** | **N** | **Min^a^** | **Max^b^** | **Mean (SD^c^)** | **P^d^** | **N** | **Min^a^** | **Max^b^** | **Mean (SD^c^)** | **P^d^** | **N** | **Min^a^** | **Max^b^** | **Mean (SD^c^)** | **P^d^** |
| *CRY1* | c.1657+3A>C | T/T | 33,998 | 23.08 | 31.51 | 27.32 (0.99) | 0.132 | 33,964 | 22.79 | 31.80 | 27.29 (1.05) | 0.163 | 32,636 | 21.50 | 33.32 | 27.42 (1.36) | 0.335 |
|  |  | T/G | 318 | 24.06 | 30.38 | 27.41 (1.00) |  | 318 | 23.54 | 30.11 | 27.38 (1.06) |  | 305 | 17.84 | 31.67 | 27.51 (1.39) |  |
|  |  | G/G | 4 | 25.08 | 28.44 | 26.5 (1.48) |  | 4 | 25.67 | 28.63 | 26.72 (1.33) |  | 3 | 22.82 | 28.08 | 26.05 (2.82) |  |

^a^Minimum; ^b^Maximum; ^c^Standard Deviation; ^d^P-value from 2-sided t-test. Homozygous carriers for the *CRY1* variant were combined with heterozygous carriers prior to performing t-tests.
