# Supplementary material for: The impact of Mendelian sleep and circadian genetic variants in a population setting
Source: PLoS Genet. 2022 Sep 22;18(9):e1010356. doi: 10.1371/journal.pgen.1010356 (PMC9499244; doi:10.1371/journal.pgen.1010356)
Supplement: S11 Table — (DOCX) [file pgen.1010356.s011.docx]

**S11 Table.** Summary statistics of sleep-midpoint estimated from accelerometer data in UK Biobank and MESA across genotype groups for variants previously reported as causal for delayed sleep phase.

|  |  |  |  | **Average for All Nights** | | | | | **Average for Weeknights** | | | | | **Average for Weekend Nights** | | | | |
| --- | --- | --- | --- | --- | --- | --- | --- | --- | --- | --- | --- | --- | --- | --- | --- | --- | --- | --- |
| **Gene** | **Variant** | **Study** | **Genotype** | **N** | **Min^a^** | **Max^b^** | **Mean (SD^c^)** | **P^d^** | **N** | **Min^a^** | **Max^b^** | **Mean (SD^c^)** | **P^d^** | **N** | **Min^a^** | **Max^b^** | **Mean (SD^c^)** | **P^d^** |
| *CRY1* | c.1657+3A>C | UKB | T/T | 33,908 | 23.37 | 30.59 | 27.01 (0.85) | 0.059 | 33,874 | 23.01 | 30.59 | 26.92 (0.91) | 0.038 | 33,022 | 21.69 | 32.68 | 27.27 (1.25) | 0.325 |
|  |  |  | T/G | 318 | 24.28 | 29.60 | 27.11 (0.88) |  | 318 | 23.51 | 29.44 | 27.03 (0.94) |  | 301 | 18.95 | 30.86 | 27.35 (1.46) |  |
|  |  |  | G/G | 4 | 25.49 | 27.38 | 26.5 (0.90) |  | 4 | 25.81 | 27.54 | 26.64 (0.73) |  | 4 | 23.25 | 27.77 | 26.25 (2.03) |  |
|  |  | MESA | T/T | 1914 | 13.19 | 34.92 | 27.05 (2.14) | 0.568 | 1,913 | 13.00 | 34.69 | 27.03 (2.01) | 0.668 | 1,904 | 13.00 | 33.92 | 27.45 (1.70) | 0.668 |
|  |  |  | T/G | 21 | 13.15 | 30.10 | 26.78 (3.32) |  | 21 | 15.59 | 29.85 | 26.84 (2.78) |  | 21 | 25.02 | 30.80 | 27.61 (1.54) |  |

^a^Minimum; ^b^Maximum; ^c^Standard Deviation; ^d^P-value from 2-sided t-test. Homozygous carriers for the *CRY1* in the UK Biobank variant were combined with heterozygous carriers prior to performing t-tests.
