# Supplementary material for: The impact of Mendelian sleep and circadian genetic variants in a population setting
Source: PLoS Genet. 2022 Sep 22;18(9):e1010356. doi: 10.1371/journal.pgen.1010356 (PMC9499244; doi:10.1371/journal.pgen.1010356)
Supplement: S12 Table — (DOCX) [file pgen.1010356.s012.docx]

**S12 Table.** P-values from burden testing of rare (MAF < 0.01%) loss-of-function and missense variants in genes outlined in this paper on self-reported sleep duration in UK Biobank.

| **Gene** | **Canonical**  **Transcript** | **Reported**  **Trait** | **Variant class** | **Sleep**  **Duration^e^** | **Sleep**  **Duration^f^** | **Sleep Duration**  **4 to 6 hours** | **Sleep Duration**  **1 to 6 hours** | **Sleep Duration**  **1 to 5 hours** | **Sleep Duration**  **1 to 4 hours** |
| --- | --- | --- | --- | --- | --- | --- | --- | --- | --- |
| *GRM1* | ENST00000361719 | FNSS^a^ | LoF^d^ | 0.872 | 0.690 | 0.556 | 0.583 | 0.183 | 0.629 |
|  |  |  | Missense | 0.413 | 0.401 | 0.907 | 0.978 | 0.964 | 0.017 |
| *NPSR1* | ENST00000359791 | FNSS^a^ | LoF^d^ | 0.431 | 0.863 | 0.647 | 0.471 | 0.022 | 0.208 |
|  |  |  | Missense | 0.445 | 0.790 | 0.560 | 0.479 | 0.493 | 0.832 |
| *ADRB1* | ENST00000369295 | FNSS^a^ | LoF^d^ | 0.204 | 0.266 | 0.340 | 0.336 | 0.458 | 0.789 |
|  |  |  | Missense | 0.150 | 0.238 | 0.076 | 0.091 | 0.390 | 0.934 |
| *DEC2/ BHLHE41* | ENST00000242728 | FNSS^a^ | LoF^d^ | 0.676 | 0.484 | 0.291 | 0.301 | 0.924 | 0.051 |
|  |  |  | Missense | 0.530 | 0.475 | 0.034 | 0.033 | 0.905 | 0.520 |
| *CRY1* | ENST00000008527 | DSPD^b^ | LoF^d^ | 0.713 | 0.898 | 0.636 | 0.580 | 0.653 | 0.353 |
|  |  |  | Missense | 0.217 | 0.292 | 0.086 | 0.072 | 0.199 | 0.583 |
| *PER3* | ENST00000361923 | FASP^c^ | LoF^d^ | 0.831 | 0.542 | 0.473 | 0.490 | 0.568 | 0.822 |
|  |  |  | Missense | 0.035 | 0.016 | 0.109 | 0.166 | 0.708 | 0.939 |
| *PER2* | ENST00000254657 | FASP^c^ | LoF^d^ | 0.522 | 0.400 | 0.909 | 0.924 | 0.101 | 0.412 |
|  |  |  | Missense | 0.819 | 0.796 | 0.610 | 0.532 | 0.299 | 0.930 |
| *CRY2* | ENST00000443527 | FASP^c^ | LoF^d^ | 0.417 | 0.611 | 0.197 | 0.192 | 0.830 | 0.661 |
|  |  |  | Missense | 0.053 | 0.085 | 0.141 | 0.161 | 0.389 | 0.687 |
| *TIMELESS* | ENST00000553532 | FASP^c^ | LoF^d^ | 0.659 | 0.871 | 0.296 | 0.336 | 0.261 | 0.715 |
|  |  |  | Missense | 0.420 | 0.365 | 0.571 | 0.439 | 0.559 | 0.789 |
| *CSNK1D* | ENST00000314028 | FASP^c^ | LoF^d^ | 0.124 | 0.042 | 0.004 | 0.004 | 0.479 | 0.807 |
|  |  |  | Missense | 0.701 | 0.659 | 0.653 | 0.624 | 0.198 | 0.053 |

^a^FNSS=familial natural short sleep; ^b^DSP=delayed sleep phase disorder; ^c^FASP=familial advanced sleep phase; ^d^LoF=loss-of-function; ^e^Sleep duration analysed on original unit scale; ^f^Sleep duration inverse-normalised prior to analysis.
