# Supplementary material for: The impact of Mendelian sleep and circadian genetic variants in a population setting
Source: PLoS Genet. 2022 Sep 22;18(9):e1010356. doi: 10.1371/journal.pgen.1010356 (PMC9499244; doi:10.1371/journal.pgen.1010356)
Supplement: S13 Table — (DOCX) [file pgen.1010356.s013.docx]

**S13 Table.** P-values from burden testing of rare (MAF < 0.01%) loss-of-function and missense variants in genes outlined in this paper on chronotype in UK Biobank.

| **Gene** | **Canonical**  **Transcript** | **Reported**  **Trait** | **Variant Class** | **Chronotype** | | **Definitely an**  **Evening Person** | | **Definitely a**  **Morning Person** | | **More or Definitely**  **an Evening Person** | | **More or Definitely**  **a Morning Person** |
| --- | --- | --- | --- | --- | --- | --- | --- | --- | --- | --- | --- | --- |
| *GRM1* | ENST00000361719 | FNSS^a^ | LoF^d^ | 0.685 | 0.508 | | 0.711 | | 0.689 | | 0.402 | |
|  |  |  | Missense | 0.676 | 0.684 | | 0.439 | | 0.733 | | 0.592 | |
| *NPSR1* | ENST00000359791 | FNSS^a^ | LoF^d^ | 0.911 | 0.363 | | 0.693 | | 0.770 | | 0.440 | |
|  |  |  | Missense | 0.686 | 0.517 | | 0.795 | | 0.884 | | 0.385 | |
| *ADRB1* | ENST00000369295 | FNSS^a^ | LoF^d^ | 0.055 | 0.349 | | 0.076 | | 0.016 | | 0.066 | |
|  |  |  | Missense | 0.682 | 0.415 | | 0.422 | | 0.419 | | 0.725 | |
| *DEC2/ BHLHE41* | ENST00000242728 | FNSS^a^ | LoF^d^ | 0.956 | 0.261 | | 0.172 | | 0.654 | | 0.981 | |
|  |  |  | Missense | 0.585 | 0.593 | | 0.933 | | 0.693 | | 0.145 | |
| *CRY1* | ENST00000008527 | DSPD^b^ | LoF^d^ | 0.326 | 0.326 | | 0.214 | | 0.541 | | 0.534 | |
|  |  |  | Missense | 0.405 | 0.096 | | 0.965 | | 0.234 | | 0.767 | |
| *PER3* | ENST00000361923 | FASP^c^ | LoF^d^ | 0.009 | 0.791 | | 0.005 | | 0.018 | | 0.007 | |
|  |  |  | Missense | 0.681 | 0.091 | | 0.021 | | 0.183 | | 0.353 | |
| *PER2* | ENST00000254657 | FASP^c^ | LoF^d^ | 1.3E-09 | 0.004 | | 3.8E-08 | | 3.4E-06 | | 5.0E-09 | |
|  |  |  | Missense | 0.345 | 0.277 | | 0.341 | | 0.811 | | 0.471 | |
| *CRY2* | ENST00000443527 | FASP^c^ | LoF^d^ | 0.015 | 0.856 | | 0.008 | | 0.085 | | 0.006 | |
|  |  |  | Missense | 0.816 | 0.492 | | 0.318 | | 0.808 | | 0.991 | |
| *TIMELESS* | ENST00000553532 | FASP^c^ | LoF^d^ | 0.512 | 0.471 | | 0.761 | | 0.926 | | 0.096 | |
|  |  |  | Missense | 0.834 | 0.250 | | 0.245 | | 0.713 | | 0.775 | |
| *CSNK1D* | ENST00000314028 | FASP^c^ | LoF^d^ | 0.199 | 0.966 | | 0.311 | | 0.154 | | 0.232 | |
|  |  |  | Missense | 0.296 | 0.150 | | 0.358 | | 0.496 | | 0.490 | |

^a^FNSS=familial natural short sleep; ^b^DSP=delayed sleep phase disorder; ^c^FASP=familial advanced sleep phase; ^d^LoF=loss-of-function.
