# Supplementary material for: The impact of Mendelian sleep and circadian genetic variants in a population setting
Source: PLoS Genet. 2022 Sep 22;18(9):e1010356. doi: 10.1371/journal.pgen.1010356 (PMC9499244; doi:10.1371/journal.pgen.1010356)
Supplement: S14 Table — There were no remaining loss-of-function carriers for GRM1, ADRB1 and CRY2 within the subset of individuals from UK Biobank who wore an accelerometer. (DOCX) [file pgen.1010356.s014.docx]

**S14 Table.** P-values from burden testing of rare (MAF < 0.01%) loss-of-function and missense variants in genes previously reported to harbour variants causal for disruptive sleep duration or timing on accelerometer estimates of sleep duration in UK Biobank. There were no remaining loss-of-function carriers for *GRM1*, *ADRB1* and *CRY2* within the subset of individuals from UK Biobank who wore an accelerometer.

| **Gene** | **Canonical**  **Transcript** | **Reported**  **Trait** | **Variant Class** | **Sleep**  **Duration^e^** | **Sleep**  **Duration^f^** | **Sleep Duration**  **4 to 6 hours** | **Sleep Duration**  **1 to 6 hours** | **Sleep Duration**  **1 to 5 hours** | **Sleep Duration**  **1 to 4 hours** |
| --- | --- | --- | --- | --- | --- | --- | --- | --- | --- |
| *GRM1* | ENST00000361719 | FNSS^a^ | LoF^d^ | NA | NA | NA | NA | NA | NA |
|  |  |  | Missense | 0.862 | 0.750 | 0.196 | 0.159 | 0.409 | 0.470 |
| *NPSR1* | ENST00000359791 | FNSS^a^ | LoF^d^ | 0.044 | 0.030 | 0.055 | 0.108 | 0.768 | 0.896 |
|  |  |  | Missense | 0.950 | 0.767 | 0.710 | 0.770 | 0.266 | 0.522 |
| *ADRB1* | ENST00000369295 | FNSS^a^ | LoF^d^ | NA | NA | NA | NA | NA | NA |
|  |  |  | Missense | 0.729 | 0.849 | 0.053 | 0.041 | 0.439 | 0.565 |
| *DEC2/ BHLHE41* | ENST00000242728 | FNSS^a^ | LoF^d^ | 0.052 | 0.053 | 0.482 | 0.478 | 0.780 | 0.926 |
|  |  |  | Missense | 0.865 | 0.722 | 0.330 | 0.311 | 0.171 | 0.559 |
| *CRY1* | ENST00000008527 | DSPD^b^ | LoF^d^ | 0.880 | 0.925 | 0.263 | 0.254 | 0.740 | 0.884 |
|  |  |  | Missense | 0.914 | 0.920 | 0.973 | 0.912 | 0.681 | 0.660 |
| *PER3* | ENST00000361923 | FASP^c^ | LoF^d^ | 0.622 | 0.590 | 0.943 | 0.883 | 0.447 | 0.625 |
|  |  |  | Missense | 0.481 | 0.517 | 0.220 | 0.192 | 0.222 | 0.235 |
| *PER2* | ENST00000254657 | FASP^c^ | LoF^d^ | 0.178 | 0.192 | 0.321 | 0.318 | 0.037 | 0.847 |
|  |  |  | Missense | 0.878 | 0.724 | 0.753 | 0.977 | 0.332 | 0.337 |
| *CRY2* | ENST00000443527 | FASP^c^ | LoF^d^ | NA | NA | NA | NA | NA | NA |
|  |  |  | Missense | 0.575 | 0.687 | 0.954 | 0.811 | 0.506 | 0.514 |
| *TIMELESS* | ENST00000553532 | FASP^c^ | LoF^d^ | 0.001 | 8.4E-04 | 0.438 | 0.341 | 0.376 | 0.752 |
|  |  |  | Missense | 0.974 | 0.779 | 0.355 | 0.198 | 0.266 | 0.512 |
| *CSNK1D* | ENST00000314028 | FASP^c^ | LoF^d^ | 0.946 | 0.931 | 0.729 | 0.709 | 0.868 | 0.918 |
|  |  |  | Missense | 0.364 | 0.368 | 0.309 | 0.286 | 0.456 | 0.646 |

^a^FNSS=familial natural short sleep; ^b^DSP=delayed sleep phase disorder; ^c^FASP=familial advanced sleep phase; ^d^LoF=loss-of-function; ^e^Sleep duration analysed on original unit scale; ^f^Sleep duration inverse-normalised prior to analysis.
