# Supplementary material for: The impact of Mendelian sleep and circadian genetic variants in a population setting
Source: PLoS Genet. 2022 Sep 22;18(9):e1010356. doi: 10.1371/journal.pgen.1010356 (PMC9499244; doi:10.1371/journal.pgen.1010356)
Supplement: S15 Table — There were no remaining loss-of-function carriers for GRM1, ADRB1 and CRY2 within the subset of individuals from UK Biobank who wore an accelerometer. (DOCX) [file pgen.1010356.s015.docx]

**S15 Table.** P-values from burden testing of rare (MAF < 0.01%) loss-of-function and missense variants in genes previously reported to harbour variants causal for disruptive sleep duration or timing on accelerometer estimates of sleep timing in UK Biobank. There were no remaining loss-of-function carriers for *GRM1*, *ADRB1* and *CRY2* within the subset of individuals from UK Biobank who wore an accelerometer.

| **Gene** | **Canonical**  **Transcript** | **Reported**  **Trait** | **Variant**  **Class** | **Sleep-midpoint^e^** | **Sleep-midpoint^f^** | **L5 Timing^g^** | **L5 Timing^h^** |
| --- | --- | --- | --- | --- | --- | --- | --- |
| *GRM1* | ENST00000361719 | FNSS^a^ | LoF^d^ | NA | NA | NA | NA |
|  |  |  | Missense | 0.910 | 0.944 | 0.277 | 0.287 |
| *NPSR1* | ENST00000359791 | FNSS^a^ | LoF^d^ | 0.586 | 0.601 | 0.682 | 0.698 |
|  |  |  | Missense | 0.968 | 0.993 | 0.480 | 0.485 |
| *ADRB1* | ENST00000369295 | FNSS^a^ | LoF^d^ | NA | NA | NA | NA |
|  |  |  | Missense | 0.015 | 0.021 | 0.945 | 0.958 |
| *DEC2/ BHLHE41* | ENST00000242728 | FNSS^a^ | LoF^d^ | 0.476 | 0.452 | 0.529 | 0.572 |
|  |  |  | Missense | 0.799 | 0.818 | 0.326 | 0.362 |
| *CRY1* | ENST00000008527 | DSPD^b^ | LoF^d^ | 0.508 | 0.544 | 0.993 | 0.970 |
|  |  |  | Missense | 0.011 | 0.009 | 0.238 | 0.233 |
| *PER3* | ENST00000361923 | FASP^c^ | LoF^d^ | 0.011 | 0.016 | 3.8E-06 | 8.6E-06 |
|  |  |  | Missense | 0.722 | 0.776 | 0.880 | 0.903 |
| *PER2* | ENST00000254657 | FASP^c^ | LoF^d^ | 5.1E-07 | 1.5E-06 | 9.2E-04 | 7.3E-04 |
|  |  |  | Missense | 0.500 | 0.551 | 0.261 | 0.277 |
| *CRY2* | ENST00000443527 | FASP^c^ | LoF^d^ | NA | NA | NA | NA |
|  |  |  | Missense | 0.382 | 0.343 | 0.729 | 0.770 |
| *TIMELESS* | ENST00000553532 | FASP^c^ | LoF^d^ | 0.143 | 0.137 | 0.016 | 0.013 |
|  |  |  | Missense | 0.971 | 0.994 | 0.988 | 0.959 |
| *CSNK1D* | ENST00000314028 | FASP^c^ | LoF^d^ | 0.767 | 0.741 | 0.397 | 0.374 |
|  |  |  | Missense | 0.623 | 0.709 | 0.735 | 0.830 |

^a^FNSS=familial natural short sleep; ^b^DSP=delayed sleep phase disorder; ^c^FASP=familial advanced sleep phase; ^d^LoF=loss-of-function; ^e^Sleep-midpoint analysed on original unit scale; ^f^Sleep-midpoint inverse-normalised prior to analysis; ^g^L5 timing analysed on original unit scale; ^h^L5-timing inverse-normalised prior to analysis.
