# Supplementary material for: The impact of Mendelian sleep and circadian genetic variants in a population setting
Source: PLoS Genet. 2022 Sep 22;18(9):e1010356. doi: 10.1371/journal.pgen.1010356 (PMC9499244; doi:10.1371/journal.pgen.1010356)
Supplement: S16 Table — (DOCX) [file pgen.1010356.s016.docx]

## S16 Table. Summary of chronotype by *PER2* loss-of-function carrier status in the UK Biobank.

|  |  | **More or definitely a morning person** | | | **Definitely a morning person** | | | **More or definitely an evening person** | | | **Definitely an evening person** | | |
| --- | --- | --- | --- | --- | --- | --- | --- | --- | --- | --- | --- | --- | --- |
| **LoF Status** | **N** | **Controls (%)** | **Cases (%)** | **P^a^** | **Controls (%)** | **Cases (%)** | **P^a^** | **Controls (%)** | **Cases (%)** | **P^a^** | **Controls (%)** | **Cases (%)** | **P^a^** |
| Non-Carrier | 170,015 | 74,360 (43.7) | 95,655 (56.3) | <0.0001 | 129,577 (76.2) | 40,438 (23.8) | <0.0001 | 113,103 (66.5) | 56,912 (33.5) | 0.0001 | 156,560 (92.1) | 13,455 (7.9) | 0.009 |
| Carrier | 64 | 9 (14.1) | 55 (85.9) |  | 27 (42.2) | 37 (57.8) |  | 57 (89.1) | 7 (10.9) |  | 64 (100) | 0 (0.0) |  |

^a^P-value derived from 2-sided Fisher’s Exact test.
