# Supplementary material for: The impact of Mendelian sleep and circadian genetic variants in a population setting
Source: PLoS Genet. 2022 Sep 22;18(9):e1010356. doi: 10.1371/journal.pgen.1010356 (PMC9499244; doi:10.1371/journal.pgen.1010356)
Supplement: S17 Table — (DOCX) [file pgen.1010356.s017.docx]

**S17 Table.** Summary of sleep-midpoint by *PER2* loss-of-function carrier status in the UK Biobank.

|  | **All Nights** | | | | | **Sleep Midpoint (Weeknights)** | | | | | **Sleep Midpoint (Weekend nights)** | | | | |
| --- | --- | --- | --- | --- | --- | --- | --- | --- | --- | --- | --- | --- | --- | --- | --- |
| **Carrier Status** | **N** | **Min^a^** | **Max^b^** | **Mean (SD^c^)** | **P^d^** | **N** | **Min^a^** | **Max^b^** | **Mean (SD^c^)** | **P^d^** | **N** | **Min^a^** | **Max^b^** | **Mean (SD^c^)** | **P^d^** |
| Non-Carrier | 34,585 | 19.45 | 30.59 | 27.01 (0.85) | <0.0001 | 34,550 | 19.22 | 30.59 | 26.92 (0.91) | <0.0001 | 33,677 | 18.92 | 32.68 | 27.27 (1.26) | 0.029 |
| Carrier | 16 | 24.04 | 27.11 | 26.05 (0.99) |  | 16 | 24.04 | 27.23 | 25.9 (1.06) |  | 15 | 25.04 | 28.21 | 26.56 (0.97) |  |

^a^Minimum; ^b^Maximum; ^c^Standard Deviation; ^d^P-value derived from 2-sided t-test.
