# Supplementary material for: The impact of Mendelian sleep and circadian genetic variants in a population setting
Source: PLoS Genet. 2022 Sep 22;18(9):e1010356. doi: 10.1371/journal.pgen.1010356 (PMC9499244; doi:10.1371/journal.pgen.1010356)
Supplement: S18 Table — (DOCX) [file pgen.1010356.s018.docx]

## S18 Table. Summary of L5-midpoint timing by *PER2* loss-of-function carrier status in the UK Biobank.

|  | **All nights** | | | | | **Weeknights** | | | | | **Weekend nights** | | | | |
| --- | --- | --- | --- | --- | --- | --- | --- | --- | --- | --- | --- | --- | --- | --- | --- |
| **Carrier Status** | **N** | **Min^a^** | **Max^b^** | **Mean (SD^c^)** | **P^d^** | **N** | **Min^a^** | **Max^b^** | **Mean (SD^c^)** | **P^d^** | **N** | **Min^a^** | **Max^b^** | **Mean (SD^c^)** | **P^d^** |
| Non-Carrier | 34,748 | 21.34 | 31.51 | 27.32 (1) | 0.027 | 34,713 | 20.09 | 31.80 | 27.29 (1.05) | 0.022 | 33,358 | 12.15 | 35.91 | 27.41 (1.43) | 0.458 |
| Carrier | 16 | 24.71 | 27.88 | 26.76 (0.85) |  | 16 | 24.71 | 28.24 | 26.69 (0.94) |  | 14 | 26.05 | 28.30 | 27.13 (0.63) |  |

^a^Minimum; ^b^Maximum; ^c^Standard Deviation; ^d^P-value from 2-sided t-test.
