# Supplementary material for: The impact of Mendelian sleep and circadian genetic variants in a population setting
Source: PLoS Genet. 2022 Sep 22;18(9):e1010356. doi: 10.1371/journal.pgen.1010356 (PMC9499244; doi:10.1371/journal.pgen.1010356)
Supplement: Attachment — Submitted filename: 20220706_PlosGenetics_Response_to_Reviewers.docx [file pgen.1010356.s020.docx]

**Comments to the Authors:**

Reviewer #1: I very much enjoyed reading the authors’ manuscript. It was nice study into the effects of previously found genetic variants linked to sleep and circadian genes. Whilst slightly limited in scope, it is nevertheless an important piece of research.
There were a few things in particular that I very much enjoyed in the paper. Mostly I found the paper easy to read and understand with only a few confusing sections (see below). Verification of the results from the main cohort (UK Biobank) in the two Finnish studies was nice to see and really helps to back up the mostly null results presented in the manuscript. Lastly the detailed sensitivity analysis was very useful in understanding the effects and showing that results presented were not caused by cherry picking of thresholds etc.
That being said I did have a few criticisms of the manuscript, these are mostly minor comments/changes.

• Sleep timing – I feel that “diurnal preference” and “chronotype” are used interchangeably here, with only the former being defined. I would either stick to one, or define both.

***We thank the reviewer for this suggestion. We have replaced “diurnal preference” with “chronotype” but used the term to define chronotype in the first instance (line 246).***

• Line 256: redundant word “tests”

***This was an error and has been removed***

• Confusing sub headings. In the Methods section subheadings are in mix of italics and underlined but no consistently. Later in the Results subheadings are just in italics. I felt that the lack of consistency was confusing.

***We thank the reviewer for pointing this out to us and apologize for any confusion this has caused. We have revised the formatting of the headers to ensure consistency throughout.***

• I don’t think that the logistic regression analyses where presented in full anywhere. As far as I could tell they were given in select cases, the full results from this for all analyses run should be presented somewhere (probably in supplemental).

***A limited number (2) of logistic regression analyses were performed for the purposes of comparisons with specific observations from previous publications. The results from these have been presented in the text and model described in the methods section.***

• Tables 1 and 2 didn’t seem very interesting to me. For the first section in the results as far as I understood the most important analysis was in Sup Table 1. I’d recommend moving T1&2 to supplemental and promoting ST1 to the manuscript.

***We agree with the reviewer and have made this change.***

• ST2: Is the 2-sided t-test correct here? If you’re looking for evidence that individuals have a short sleep, should it not be a 1-sided test? I think similar arguments can be made for later tables as well.

***We understand the reviewer’s point of view, but 1-sided tests can be viewed with suspicion and so we have used the standard 2-sided test. None of the results become significant if using a 1-sided test.***

• Line 347: “We previously tested the …” Is this missing a reference here? Likewise on Line 363 “has previously been associated with…” also seems to missing a reference. Either needs references adding in or these sentences need removing.

***We have now added the reference to Jones et al. (line 383) and Patke et al (line 417).***

• Table 5 seems to show an association between sleep midpoint and the CRY2 variant in the accelerometer data, but this isn’t mentioned in the text?

***Although CRY2 has a P-value = 0.04 in Table 5 (now Table 4), the effect is 42 minutes later – not earlier as would expected for advanced sleep phased. In addition, this p-value does not pass Bonferroni statistical significance after accounting for multiple testing. We have therefore not included this in the text.***

• ST5: Is the “definitely a morning person” section of this table the same as the “definitely a morning person” section of Table 5? If so then why are the Finnish values different in these two tables? Also the p<0.0001 should match the p<0.001 in Table 5 for consistency.

***We thank the reviewer for highlighting this. ST5 (now ST6) Table 5 (now Table 4) are now consistent after updating the supplementary table with the finalised results from the Finnish studies (main table stays the same).***

• I would switch the order of ST6 and ST7 (and likewise the order of ST9 and ST10) to match the ordering in Table 5 (and Table 6).

***We have switched the relevant supplementary tables as suggested.***

• I think that it’s worth mentioning the observed differences between weekday and weekend nights in ST9

***We have added this observation to lines 426-427.***

• Supplemental Figures 1-3 add nothing to the manuscript as they are not referred to (just tacked on to line 374). They should be removed.

***We agree with the reviewer and have removed these from the manuscript.***

Reviewer #2: This is a straightforward publication that helps clarify the literature. This is an important and well-done publication. Non-replications, including from the same author’s lab where the work has been initially generated, are important as it is harder and harder to make sense of the literature.

The authors show lack of replication of large effects of 8/10 previously published “mendelian circadian and sleep variants” reported in single or rare families. The authors also extend the study of these genes by conducting burden tests, often finding minimal or no effects, except for PER2 and PER3. It is then argued that out of all 10 mutations previously reported maybe only one, S662G, not found in this sample, maybe pathogenic. The other previously published mutation not found in this survey was NPSR1 Y206H.

I have a few comments.

It would be useful to list in the abstract the two out of 10 mutations that were not found (NPSR1 Y206H and PER2 S662G).

***We have amended the abstract to provide this information from the outset.***

The publication focusses mostly on circadian and short sleep variants, so the title is misleading. For example, a new mutation g.42184347T>C; p.Lys68Arg; rs537376938 in the cleavage site of HCRT has been recently reported to be associated with idiopathic hypersomnia (minor allele frequency of 1.67% in cases versus 0.32% in controls, P = 2.7 × 10-8, odds ratio = 5.36) characterized by sleepiness and excessive sleep in Asians (Miyagawa et al. NPJ Genom Med. 2022 Apr 12;7(1):29). This was somewhat surprising as a mouse model with a truncated form of pre-proorexin generating only orexin A, even when homozygous, has no phenotype. Miyagawa et al. reports in the publication that the variant is present in non-Finnish Europeans at 0.013%, so most likely it would be present in the UK biobank analyzed here. Perhaps analyzing this variant should be confirmed and included, as the UK biobank has questions on excessive sleepiness and sleep time. In this case, the title would be more accurate.

***We understand the reviewer’s point. However, there is only 1 carrier in the European ancestry individuals in the UK Biobank. We also feel this is a slightly different situation to the other variants included here as this variant was identified in a case control setting and is a risk factor rather than being described as a monogenic familial cause. We have therefore not included this in the manuscript.***

The main issues that could have led to a difference in results include: 1) ascertainment of phenotypes, 2) amount of cases/nature of pedigree (the more polygenic a trait is the faster genetic risk decreases from one generation to the next) and 3) genetic background/ethnicity (even so MESA was included, it is a small sample). These limitations are not all well discussed. Even in the small number of cases identified with some of these mutations, a PCA analysis could reveal unsuspected population stratification or founder effects. Has this been looked at?

***We appreciate the reviewer’s comments. We have covered the ascertainment bias issue in paragraph 2 of the discussion and we have referenced our previous work on the topic of ascertainment bias in monogenic disease. We address the issue of sample size at the end of paragraph 5 where we highlight that for most variants reported we have substantially more variants in our study than in the published studies. The issue of genetic background and ethnicity is discussed in paragraph 3, where we describe how our work on GWAS has identified hundreds of polygenic variants influencing sleep and circadian measures and that this may well influence the penetrance of a “monogenic” variant.***

***We have been stringent in our inclusion of only unrelated Europeans using PCA analysis. We also think that the most likely result of unadjusted population stratification would be false positive associations, rather than the lack of association observed here. For the benefit of the reviewer we have provided a PCA plot of all UK Biobank participants, highlighting the unrelated Europeans with exome-sequence data included in our analyses.***

***Fig 1. PCA of all UK Biobank samples. Unrelated individuals of EUR ancestry included (orange) and excluded (blue) from analyses highlighted***

The fact many of these variants had thorough functional characterization (often in mouse) that was used as evidence of involvement is not discussed at all. The authors need to discuss briefly the limits of functional characterization. Indeed, it is argued that PER2 S662G has sufficient evidence for being pathogenic. Indeed, Weeldon et al show here that haploinsufficiency of PER2 is associated with a morning phenotype. PER2 knock out are also phase advanced (Nature; 1999 Jul 8;400(6740):169-73). Finally, for S662G, there is multiple in vitro evidence linking the S662 phosphorylation site with casein kinases (although the exact mechanism is somewhat debated see Cell. 2007 Jan 12;128(1):22-3. doi: 10.1016/j.cell.2006.12.024), other key circadian genes, plus the effects of S662G and S662A were assessed in mice. This is reasonable.

However, there is also similar rodent evidence be written for other mutations that was used to “confirm” the phenotype that was not confirmed in this study such 1) the T44A in the human CKIδ gene had a lot of experiment support; 2) the A260T of CRY2 which is in its FAD binding domain (postulated to increase the affinity of FAD for the E3 ubiquitin ligase FBXL3, thus promoting its degradation). 3) the Patke et al. CRY1 DSPD mutation, located in the 5′ splice site of exon 11 and leading to exon 11 and an in-frame deletion of 24 residues in the C-terminal region of CRY1. This was postulated to lead to an enhanced affinity of this repressor for the circadian activator proteins CLOCK and BMAL1, which could lengthen the period of circadian molecular rhythms. These also all had reasonable explanations for the phenotype observed. This raises questions on the value of functional characterizations done in mouse or other models that should be briefly mentioned.

Similarly, NPSR1 Y206H which is not found is not discussed. Is there something special about the NPSR1 variant or this gene?

***We agree with the reviewer. One of the main issues with the original studies is that while there was extensive animal model and in vitro functional data presented in each of the papers the human genetic evidence is relatively weak and therefore the relevance in human populations can be unclear. We have therefore added the highlighted sentence to the following paragraph:***

*The functional effect of each the variants assessed here are supported by extensive in vitro and animal model studies. The level of human genetic evidence varies across studies, from 6 individuals from a single pedigree for the ADBR1 variant to 78 individuals from 7 families for the CRY1 c.1657+3A>C variant.* ***While functional evidence in animal models is important to understand the biology of the associated variants, it is important to demonstrate robust human genetic evidence to ensure relevance in humans.*** *It is therefore possible that some of the reported variants with weaker human genetic evidence do not cause monogenic sleep and circadian conditions in humans.*

***For the NPSYR1 variant we can’t comment specifically about this gene because of the rarity and inability to analyse this variant in the data we have access to.***
